# Antimicrobial Potential of Conjugated Lignin/Morin/Chitosan Combinations as a Function of System Complexity

**DOI:** 10.3390/antibiotics11050650

**Published:** 2022-05-12

**Authors:** Zvezdelina Yaneva, Georgi Beev, Nikolina Rusenova, Donika Ivanova, Milena Tzanova, Daniela Stoeva, Monika Toneva

**Affiliations:** 1 Chemistry Unit, Department of Pharmacology, Animal Physiology, Biochemistry and Chemistry, Faculty of Veterinary Medicine, Trakia University, Students Campus, 6000 Stara Zagora, Bulgaria; donika.ivanova@trakia-uni.bg (D.I.); monika_ra4eva@abv.bg (M.T.); 2 Department of Biochemistry, Microbiology and Physics, Faculty of Agriculture, Trakia University, Students Campus, 6000 Stara Zagora, Bulgaria; gbeev@abv.bg (G.B.); milenatzanova@hotmail.com (M.T.); dck2004@abv.bg (D.S.); 3 Microbiology Unit, Department of Veterinary Microbiology, Infectious and Parasitic Diseases, Faculty of Veterinary Medicine, Trakia University, Students Campus, 6000 Stara Zagora, Bulgaria; n_v_n_v@abv.bg; 4 Department of Chemistry and Biochemistry, Faculty of Medicine, Trakia University, 11 Armeiska Str., 6000 Stara Zagora, Bulgaria

**Keywords:** morin, chitosan, lignin, antibacterial activity, combined systems

## Abstract

As natural biopolymers, chitosan and lignin are characterized by their good biocompatibility, high biodegradability and satisfactory biosafety. The active polymers’ functional groups are responsible for the potential of these biomaterials for use as carrier matrices in the construction of polymer–drug conjugates with prospective applicability in the fields of medicine, food and agriculture—subjects that have attracted attention in recent years. Hence, the aim of this research was to place substantial emphasis on the antimicrobial potential of flavonoid–biopolymer complex systems by assessment of the probable synergetic, additive or antagonistic effects arising as a function of systemic complexity. The joint implementation of morin, chitosan and lignin in conjugated two- and three-component systems provoked species-dependent antimicrobial synergistic and/or potentiation effects against the activity of the tested bacterial strains *Staphylococcus aureus* ATCC 25923, *Escherichia coli* ATCC 25922, *Pseudomonas aeruginosa* ATCC 27853 and the clinical isolate *Bacillus cereus*. The double combinations of morin–chitosan and morin–lignin resulted in a 100% increase in their inhibitory activity against *S. aureus* as compared to the pure biocompounds. The inhibitory effects of the three-component system, in decreasing order, were: *S. aureus* (IZ = 15.7 mm) > *P. aeruginosa* (IZ = 15 mm) > *B. cereus* and *E. coli* (IZ = 14 mm). All tested morin-containing two- and three-component systems exhibited clear and significant potentiation effects, especially against *S. aureus* and *B. cereus*. The results obtained are a prerequisite for the potential use of the studied conjugated lignin–morin–chitosan combinations in the construction of novel drug-carrier formulations with improved bioactivities.

## 1. Introduction

According to recent studies, the pathogenesis of many diseases involves various factors, which shifts the focus from the development of new drugs implementing the conventional “one target–one drug” model to a new “multitarget–multidrug” model [1]. 

Clarifying the additive, synergistic or antagonistic effect of the application of multicomponent systems containing combinations of biologically active substances requires the development of strategies involving several successive stages: (i) determination of the pharmacological activity of the individual compounds; (ii) clarifying the interactions between them; (iii) determining a combination of dominant compounds based on an analysis of their respective contributions to the activity; and (iv) assessment of the biological activity of the combination [1,2].

Modern scientific literature provides a large number of studies on the synergistic and additive activity of the combined use of natural polyphenols, polyphenols and vitamins, and polyphenols and antibiotics [3,4,5]; however, only a limited number of studies have addressed the complex application of combinations of flavonoids and the biopolymers chitin and lignin. 

Aside from its abundance and low cost, the favorability of lignin is due to its numerous attractive properties, such as biocompatibility, biodegradability, environmental amiability, low toxicity, antioxidant activity, high carbon content and high thermal stability [6]. However, recent research has provided only limited data on the antibacterial, antiviral and antitumor activities of different types of lignins, which are prerequisite for the potential biomedical applications in which there is growing interest [7,8,9]. It was found that lignin extracts showed considerable antimicrobial activity against *Listeria innocua* [9], while, according to Tran et al. [10], lignin proved to be less effective against *Escherichia coli* as compared with *Staphylococcus aureus*.

A natural, effective and inexpensive hindered phenolic antioxidant mixture was prepared by blending lignin into quercetin [11]. The reported antioxidant efficacy of the combination, the reduced extent of polymer aggregation and the lignin potential of newly formed conjugated structures indicated that the addition of lignin to the flavonoid could provide a green alternative to the expensive quercetin and synthetic antioxidants used in food, cosmetics and pharmaceutical technologies [9,10,11]. 

The important features of lignin can be synergistically combined with the advanced functionalities of well-defined polymers, such as gelatin, chitosans, hyaluronic acids, etc. [12,13]. Due to the valuable physicochemical properties (a number of reactive functional groups, gelation and significant sorption potential) and the essential bioactivities (biodegradability, biocompatibility, antibacterial, antifungal and antitumor potential) of chitosan, scientific interest in and expectations for the design and successful biomedical application of chitosan-based hybrid systems are progressively increasing [14,15,16]. The addition of chitosan to lignin films enhanced their activity against both Gram-positive and Gram-negative bacteria [12]. A newly developed multifunctional antibacterial, antioxidant and injectable cryogel formed by combining lignin with gelatin displayed satisfactory antimicrobial activity against surgical site infection-associated pathogenic bacteria. Furthermore, lignin–gelatin cryogel was characterized by free radical scavenging potential and sufficient cytocompatibility [17].

A substantial emphasis on the antimicrobial potential of flavonoid–biopolymer complex systems by assessment of the probable synergetic, additive or antagonistic effects arising as a function of systemic complexity is of utmost significance for the design of polymer–drug conjugates with prospective applicability in the fields of biomedicine, food and agriculture.

The conducted literature survey revealed that the recent scientific literature does not contain studies on the antimicrobial activity of the two- and three-component combinations of the flavonoid morin and the polymers lignin and chitosan in various qualitative and quantitative ratios. The latter is associated with a lack of clearly defined strategies for the detailed clarification of the individual role of each of the biocomponents in the complex formulations and in-depth comparative analysis of the potentially arising synergistic, additive or antagonistic antimicrobial effects of the studied systems, and it is this deficiency that provoked the present study and determined its purpose and objectives.

The aim of the study was to conduct comparative in vitro studies of the antimicrobial activity of two- and three-component systems containing the natural plant flavonoid morin, the heteropolysaccharide chitosan (CH) and the complex biopolymer lignin (LGN) in various qualitative and quantitative combinations and to clarify the effect of the individual components on the overall bioactivity of the complex systems.

## 2. Results and Discussion

### 2.1. Comparative Analyses of the Antimicrobial Potential of Single-, Two- and Three-Component Lignin–Morin–Chitosan Systems

*S. aureus* and *E. coli* are the most common causes of surgical site infection, accounting for 18.8 and 17.3% of clinical infections requiring hospitalization, respectively [18]. Antimicrobial tests of the studied single-, two- and three-component systems against the Gram-positive and Gram-negative bacteria were performed using an agar diffusion method, according to our previously developed protocol. The experimental results are presented in Table 1. Obviously, no inhibition zones against these pathogens were observed for the pure flavonoid morin within the entire examined concentration region ranging from 333.33 µg/mL to 1000 µg/mL. 

**Figure 1:**
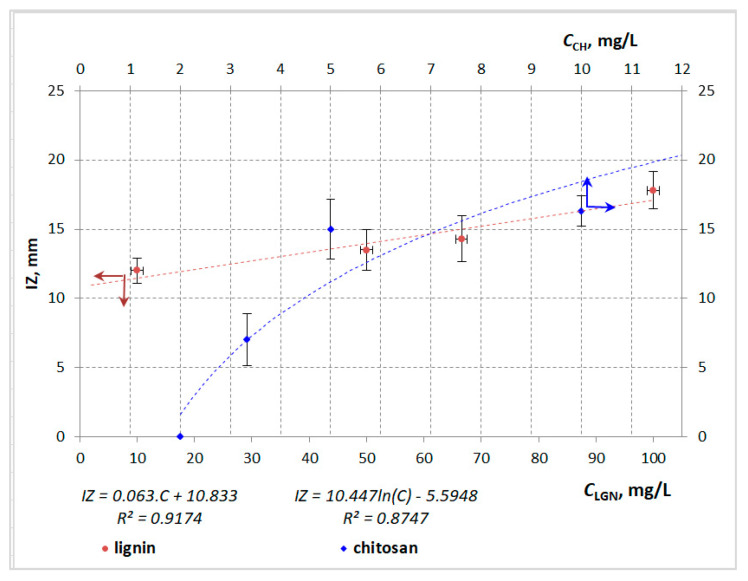
Dependence between the antimicrobial activity of chitosan and lignin (expressed as IZ, mm) and the biopolymer concentrations against *S. aureus*.

Both pure biopolymers, however, exhibited satisfactory antimicrobial activity against *S. aureus* in a dose-dependent manner—linear dependence in the case of lignin and an exponential mode in that of chitosan, with satisfactory values for the correlation coefficients (R^2^) (Figure 1). Additionally, chitosan was characterized by higher activity than lignin when applied at the same concentration (10 mg/mL). The heterobiopolymer inhibited the growth of *P. aeruginosa* when applied in higher concentrations (5–10 mg/mL). No activities of the polymer materials against *B. cereus* and *E. coli* were registered.

**Figure 2:**
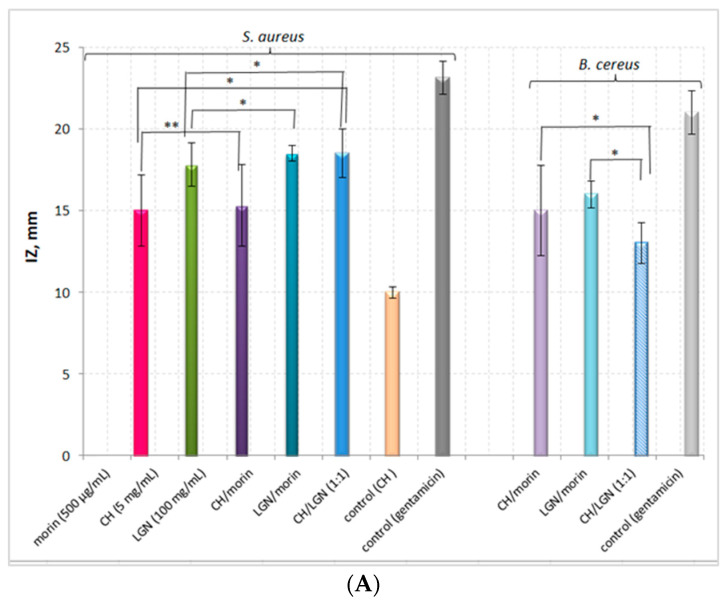

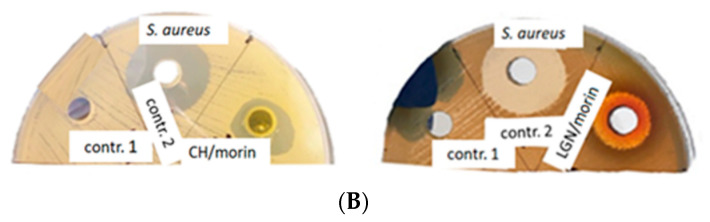
Antimicrobial potential of single- and two-component morin–biopolymer, biopolymer–biopolymer systems against *S. aureus* and *B. cereus*. (Statistical significance of the experimental results: * *p* < 0.005; ** *p* = 0.073.): (**A**) Graphical representation of the experimental data, (**B**) Photos of the inhibition zones against *S. aureus*.

The addition of morin to chitosan did not result in any significant increase in the antimicrobial activity of the system against *S. aureus* as compared to the pure polymer, while the inhibition zone of the lignin–morin system was increased by 3.8% as compared to that of pure lignin against the same bacterial strain (Figure 2). Interestingly, the two-component chitosan–morin system did not exhibit any antibacterial potential against *P. aeruginosa*, despite the registered activity of the pure biopolymer, which is an indication of a probable antagonistic effect between the flavonoid and the polymer against the Gram-negative strain. The antimicrobial activity of the CH–LGN system surpassed that of the single-component suspensions, which is an indication of a synergistic effect arising from the combined application of both biopolymers against *S. aureus*. The potential of the combination LGN–morin against *S. aureus* was approximately 22% higher than that of CH–morin. 

The behaviour of both two-component systems against the Gram-positive *B. cereus*, however, provoked great interest, as their antimicrobial potentials were commensurable with those against *S. aureus*, considering the absence of inhibitory effects when the three pure substances were used against this strain. Consequently, the double combination of the natural polyphenol morin with each of the biopolymers resulted in a 100% increase in their inhibitory activity as compared to the pure biocompounds. 

**Figure 3:**
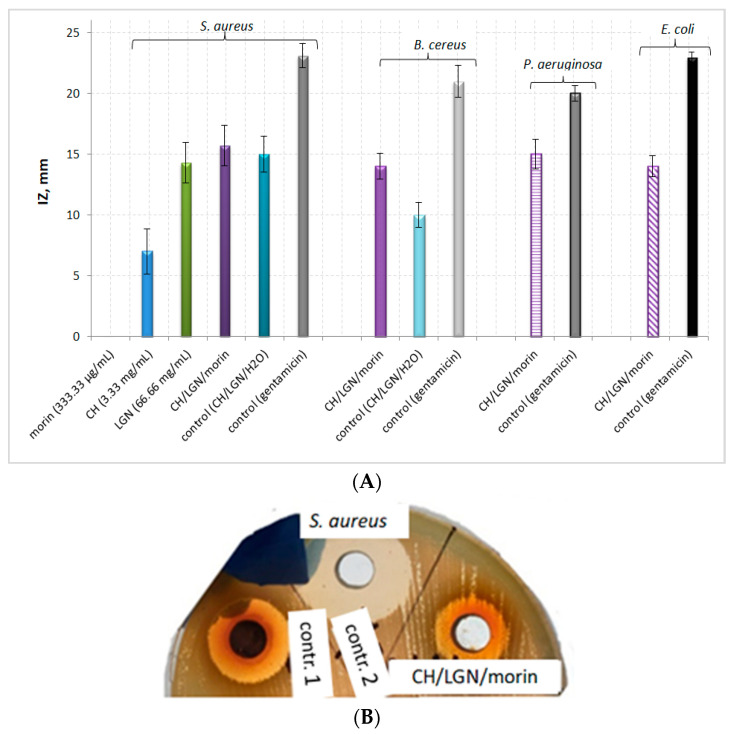
Antimicrobial potential of the three-component morin–chitosan–lignin system against *S. aureus*, *P. aeruginosa*, *B. cereus* and *E. coli*. (All experimental results were statistically significant: *p* < 0.005.): (**A**) Graphical representation of the experimental data; (**B**) A photo of the inhibition zones against *S. aureus*.

The antimicrobial potential of the triple combination morin–lignin–chitosan was also investigated (Table 2, Figure 3). The comparative analyses of the experimental results yielded several significant findings. Firstly, the three-component system displayed antimicrobial potential against the four tested bacterial strains despite the lack of potential of the two-component systems against both Gram-negative strains. Secondly, the antibacterial activity of the system against *S. aureus* increased by 55% compared to pure chitosan and by 6.4% compared to pure lignin. The inhibitory effect of the three-component system decreased in the order: *S. aureus* (IZ = 15.7 mm) > *P. aeruginosa* (IZ = 15 mm) > *B. cereus* and *E. coli* (IZ = 14 mm). No clear dependence between the type of microbial strain and the antimicrobial potential of the three-component system was observed.

As the initial concentrations of the bioactive compounds in the three-component system were lower than those in both two-component formulations, a direct comparative assessment of their antimicrobial activities based on inhibition zones could not be conducted. However, the approximately equal values of the inhibition zones (IZs) for CH–morin and CH–LGN–morin were an obvious indication of the enhancement of the antimicrobial capacity of the three-component system. Thus, undoubtedly the combination of the flavonoid with both biopolymers in a conjugated formulation leads to an improvement in its activity against *S. aureus*. The single-component systems did not display antimicrobial activity against *B. cereus*, but the potential of the two- and three-component systems was commensurable or slightly lower than that against *S. aureus*.

**Figure 4:**
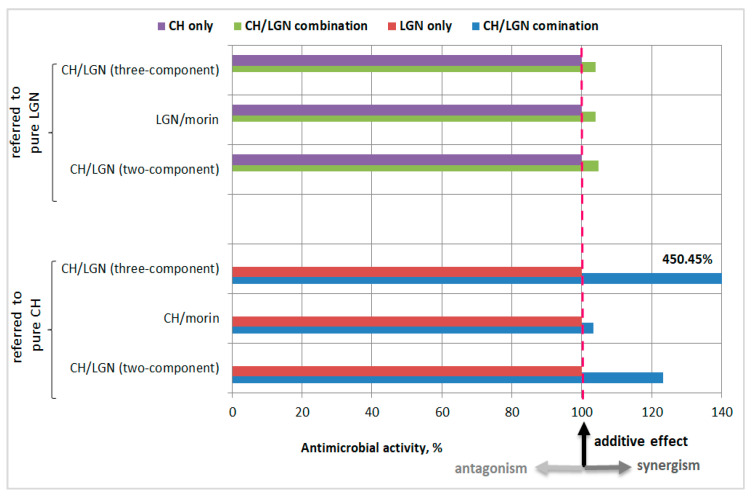
Effects of CH and LGN combinations on *S. aureus* inhibition as a percentage of the effect of both biopolymers applied alone.

To determine the effect of the combined application of CH and LGN as a two-component system and within the three-component system with morin, their antibacterial activity was calculated as a percentage of the effect of the respective biopolymers applied alone (Figure 4). The results located to the right of the reference dashed line indicate synergism in the combination CH–LGN relative to CH alone and LGN alone. The most significant synergistic effect (450.45%) was observed when lignin (with a final concentration of 66.6 mg/mL) was added to chitosan (with a final concentration of 3.33 mg/mL) as compared to chitosan alone (3.33 mg/mL), followed by the synergism (120.33%) arising from the addition of lignin (with a final concentration of 100.00 mg/mL) to chitosan (with a final concentration of 5.00 mg/mL) as compared to chitosan alone (5.00 mg/mL). The latter results are denoted with blue bars in Figure 4. The experimental data displayed commensurable synergistic effects arising from the addition of chitosan to lignin (green bars) as compared to lignin alone. Obviously, neither additive (coinciding with the reference line) nor antagonistic (to the left of the reference line) effects were observed. 

Considering the experimental results presented in Table 2, it could be concluded that all tested morin-containing two- and three-component systems exhibited clear and significant potentiation effects, as the flavonoid did not elicit a response on its own but showed enhanced responses in combinations with CH, LGN and CH–LGN, especially against *S. aureus* and *B. cereus*.

### 2.2. Structure–Activity Relationships: Effects of Flavonoid–Biopolymer, Biopolymer–Biopolymer and Flavonoid–Biopolymer–Bacteria Interactions on the Antimicrobial Activities of the Studied Conjugated Combinations

To reveal the probable interactions between morin, chitosan and lignin when applied in double or triple combinations and to explain the experimentally determined synergistic effects and antimicrobial potential against the tested Gram-positive and Gram-negative bacterial strains of the combined systems and to elucidate the mechanisms provoking such activities, it was necessary to encompass and analyze in detail the biofunctionalities of the single compounds and the complex systems in terms of their molecular structural characteristics and the nature of the bacterial strains, in light of novel scientifically significant studies. 

Gram-positive bacterial cell walls include a thick peptidoglycan layer containing teichoic acids, which are responsible for the negative charge of the bacterial surface. Actually, teichoic acids are one class of “responsibles” for Gram-positive bacterial resistance to antimicrobial agents, and their attached substituents regulate the negative charge of the bacterial cell, which also prevents the binding of extracellular molecules. The strong negative charge of Gram-negative bacterial cell walls is due to the outer membrane lipopolysaccharide layer [19]. 

**Figure 5:**
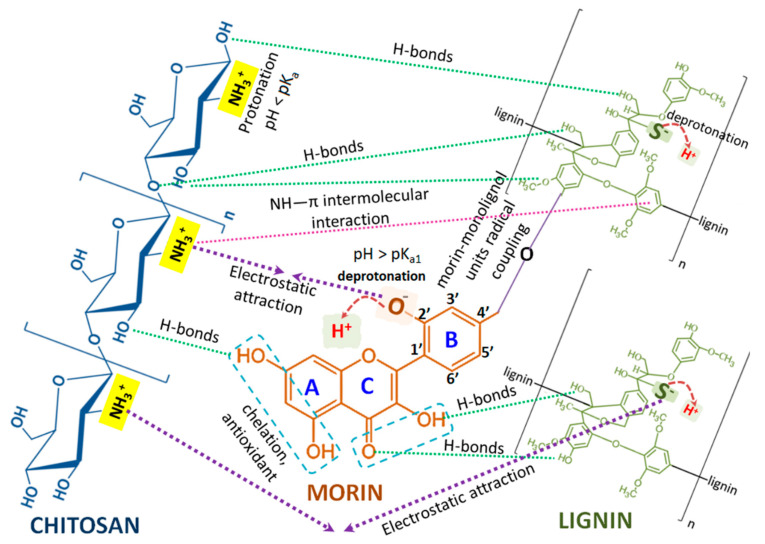
Scheme of the possible intermolecular interactions between morin, chitosan and lignin.

As a natural flavonoid, morin possesses five phenolic OH-groups, a keto (>C=O) group and an O-heteroatom in its molecule which determine its capability of forming non-covalent multiple hydrogen bonds, hydrophobic interactions and van der Waals attractions with proteins and other biomolecules, as well as forming covalent bonds with cell surface adhesion proteins and cell wall polypeptides, e.g., between the carbonyl groups of flavonoids and the thiol (–SH) and amino groups of amino acids (Figure 5). Scientific studies have proposed that the antimicrobial activity of plant flavonoids is associated with their influence on the fluidity of internal and/or external bacterial membranes causing nullification of the membrane potential and a decrease in ATP production and cell motility [18]. 

According to the scientific data, the interaction of chitosan with teichoic acids in *S. aureus* cell walls provoked inhibition of bacterial growth up to approximately 100% [20]. The biopolymer also damaged *E. coli* cell membranes, which provoked extracellular leakage of DNA and proteins from the cytoplasm. The latter observation was proved by SEM analyses revealing serious alterations to the outer membrane of chitosan-treated *E. coli* cells as well as coagulated cytosolic components [21,22]. 

Lignin is a heterogeneous and recalcitrant polymer with a polyphenolic structure formed by the polymerization of monolignols (coniferyl, sinapyl and p-coumaryl alcohols) by radical coupling reactions, generating guaiacyl, syringyl and hydroxyphenyl units with the three-dimensional molecular architecture of the natural polymer [23]. The diversity of linkages between the monolignol moieties combined with the variety of O-containing functional groups (phenolic, methoxy, ethoxy, carbonyl) explain the antimicrobial potential and other bioactivities of lignin [24].

**Figure 6:**
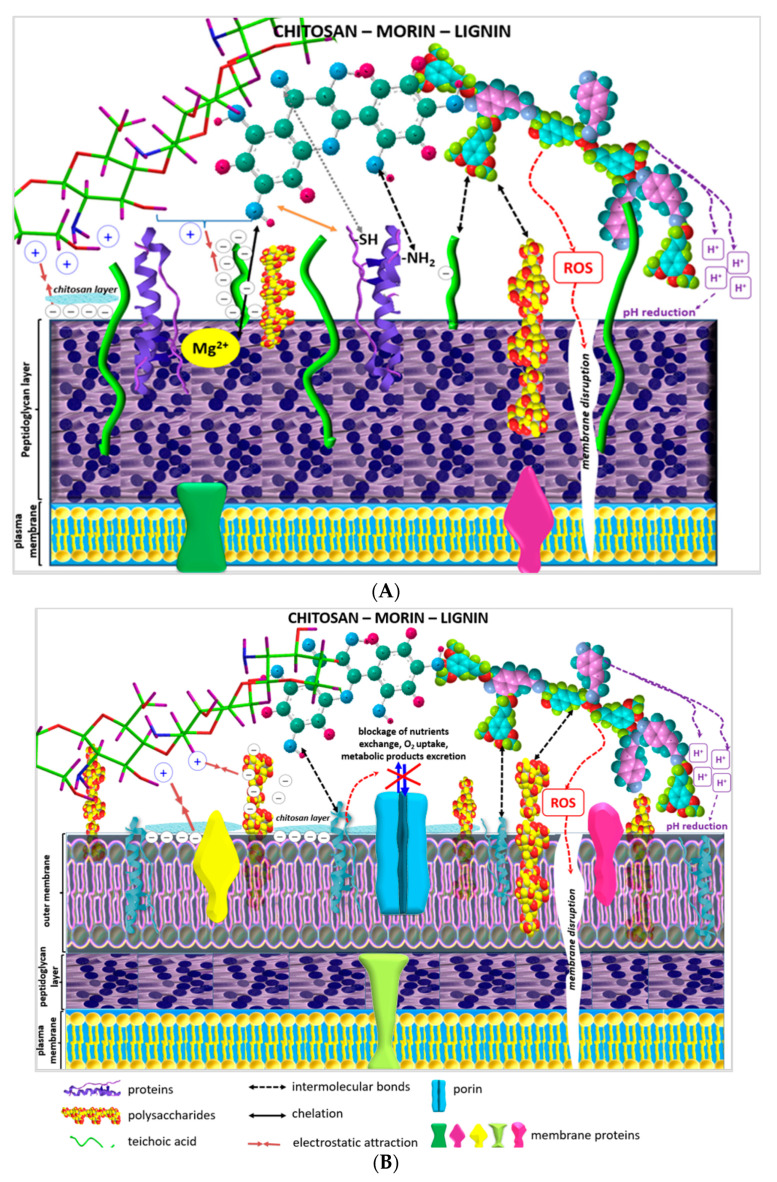
Schematic representation of the postulated antimicrobial activity mechanisms of the studied two- and three-component morin–chitosan–lignin combined systems against: (**A**) Gram-positive and (**B**) Gram-negative bacteria.

An increase in pH leads to an increase in the net negative charge of lignin due to intensified deprotonation of phenolic –OH groups, which, in turn, results in increased concentrations of H^+^, reducing the pH on the surface of the heteropolymer. The alkali lignin used in the present study was dissolved in distilled water, so the solution had an alkaline pH and, consequently, a high negative charge. Thus, electrostatic repulsion forces between polymers and negatively charged bacterial membranes are expected to occur. On the other hand, however, when the proton concentration is high, acidic conditions on the bacterial cell surface prevail and the proton dynamics of cell membranes could be destroyed (Figure 6A,B) [25].

Ning et al. associated the antibacterial activity of lignin with membrane disruption and increased levels of intracellular oxidative stress in Gram-positive bacteria [26]. These stressful environment conditions could induce ROS production which, in turn, could cause the destruction of bacterial structures and intracellular mediated functions and lead to ROS-mediated bacterial apoptosis [8,24,26]. According to another study, the established antibacterial potential of lignins against S. aureus and E. coli was due to the interaction of the heterobiopolymer carbohydrates with the bacterial cell wall involving the generation of localized heat and reactive oxygen species (ROS). The selective activity of lignins towards different bacterial types was also determined [9,27]. 

In our study, as stated earlier, the pH of the morin–chitosan suspension was <7 due to the fact that the flavonoid was dissolved in EtOH:PBS (pH = 4.6) and the biopolymer in 1% lactic acid. Thus, regarding the chemical structure of both bioactive compounds and their behavior in acidic media, the existence of intermolecular H-bonds between morin OH-groups and chitosan OH- and NH_2_- groups is highly presumptive (Figure 6) [28]. Furthermore, as the pH was higher than the dissociation constant of the most acidic 2′–OH flavonoid group (pK_a1_ ≈ 3.5), it was deprotonated. Considering that the pK_a_ value of chitosan varies between 6.17 and 6.51, obviously at a pH = 4.6 the biopolymer amino groups would have been protonated. Consequently, strong electrostatic attractional forces between the NH_3_^+^ groups of chitosan macromolecules and morin 2′-O phenoxide anions occurred [29]. 

The formed two-component morin–chitosan conjugated system was electrostatically attracted by the outer membrane negatively charged lipopolysaccharides of Gram-negative bacteria and teichoic acids of Gram-positive bacteria, leading to combinations of the following proposed effects: blockage of intra-/extracellular exchanges, alterations of cell wall rigidity, cell wall disruption, leakage of cytoplasmic content and eventual entry into the cell (Figure 6A,B) [22,30]. 

With respect to the structural characteristics of the cell walls of Gram-positive bacteria, it is well-known that teichoic acids, as highly negatively charged polymers, can also participate in cation-sequestering mechanisms, binding Ca^2+^ and Mg^2+^ for their eventual transport into the cell. Thus, another possible mechanism outlining the satisfactory antimicrobial potential of the morin–chitosan combination is a process of complexation of the chelating part of the flavonoid molecule with the Mg^2+^/Ca^2+^ ions in the cell walls of Gram-positive bacteria (Figure 6A) [31]. 

Since porins, as beta barrel proteins, in the Gram-negative bacterial membrane are responsible for nutrient uptake, serving as pores that allow passive diffusion, possible blockage of nutrient exchange, oxygen uptake and/or excretion of metabolic products formed by chitosan, polymer layer deposition on the cell surface and subsequent cell death of aerobic bacteria is another potential mechanism of the biopolymer’s antibacterial activity (Figure 6B) [22,31,32]. 

The intermolecular interactions between lignin and morin are due to the formation of morin–monolignol units by a mechanism of radical coupling (Figure 6) [33]. The hydroxyl group present in the phenolic ring of alkali lignin can interact with morine phenolic groups, β-1,4-glycosidic oxygen and the OH- groups of chitosan. A weak bond between chitosan OH- and lignin CH_3_O- groups, as well as intermolecular interactions between the aromatic rings of alkali lignin and the secondary -NH_2_ groups of chitosan, have also been possible [34]. It was postulated that lignin adhesion to the bacterial membrane could be supported by the presence of carbohydrate moieties in lignin structures [9,35,36]. 

In conclusion, although the precise mechanism of action of the bioflavonoid and both biopolymers against Gram-positive and Gram-negative pathogenic microbial cells has not been completely defined, numerous studies have presented similar results. Therefore, the hypothetical mechanisms by which chitosan–morin–lignin combined systems can inhibit microbial growth include: (i) electrostatic interactions between microbial cell surfaces causing cell wall disruption and intracellular component leakage; (ii) adhesion and penetration of the morin–polymer combinations into the cell membrane imposing sequential negative effects on protein synthesis processes; (iii) chelation of fundamental nutrients and essential metals; (iv) ROS production and cell surface pH reductions resulting in mediated bacterial apoptosis. 

## 3. Materials and Methods

### 3.1. Chemicals 

The reagents—lignin (alkali, CAS No: 8068-05-1), chitosan (medium molecular weight, CAS No.: 9012-76-4), morin (C_15_H_10_O_7_, purum, CAS: 654055-01-3), ethanol (EtOH, p.a. ≥ 99.8%), NaOH (p.a., HPLC), CH_3_COOH (p.a., HPLC), HCl (ACS reagent, 37%), phosphate-buffered saline (PBS, P-3813) and gentamicin solution (CAS No: 1405-41-050 mg/mL in deionized water, liquid, 0.1 μm filtered, BioReagent, suitable for cell culture)—were supplied by Sigma-Aldrich (St. Louis, MA, USA). Lactic acid (CH_3_CH(OH)COOH) (80%) was supplied by Chimtex, Bulgaria.

### 3.2. Flavonoid–Chitosan–Lignin Systems 

The compositions and concentrations of the studied single-, two- and three-component systems, as well as the respective negative controls, are presented in Table 2.

### 3.3. Antimicrobial Screening 

Agar well diffusion method was used to screen the antibacterial activity of the studied systems against *Staphylococcus aureus* ATCC 25923, *Escherichia coli* ATCC 25922, *Pseudomonas aeruginosa* ATCC 27853 and the clinical isolate *Bacillus cereus*, according to our previously reported protocol [37]. The volume of the samples added to the wells was 100 µL. A positive control with gentamicin at a concentration of 12.5 µg/mL was performed. The plates were incubated at 370C for 24 h under aerobic conditions. Antimicrobial activity was detected by measuring the inhibition zone (IZ, mm) (including the well diameter) that appeared after the incubation period [38]. 

### 3.4. Statistical Analysis 

The data obtained from the antimicrobial studies were expressed as means ± standard deviation (SD) from three repetitions. Statistical significance was determined using XLStat Microsoft Excel 2010 (Microsoft Corporation, WA, USA). A value of *p* < 0.05 was considered statistically significant.

## 4. Conclusions

The joint implementation of morin, chitosan and lignin in conjugated two- and three-component formulations resulted in antimicrobial synergistic and potentiation effects against the tested Gram-positive (*S. aureus, B. cereus*) and Gram-negative (*E. coli, P. aeruginosa*) bacterial strains, which are prerequisite for their potential application in the construction of novel drug-carrier formulations with improved bioactivities. Based on the detailed discussion of the probable intermolecular interactions between the plant flavonoid morin and both biopolymers (chitosan and lignin), as well as their single antibacterial potential, in summary, the overall diverse and complex mechanisms of the antimicrobial performances of the double and triple systems were found to encompass various activity pathways, including: bacterial cell wall targeting, lipid membrane disruption and membrane receptor and ion channel blockage, provoking obstruction of bacterial metabolite excretion and nutrient exchange and causing subsequent pathogenic bacterial death. 

## Figures and Tables

**Table 1:** Antimicrobial activity of the studied single-, two- and three-component systems and the respective negative and positive controls against the four microbial strains detected by measuring the inhibition zone (IZ, mm).

System	*S. aureus*	*B. cereus*	*P. aeruginosa*	*E. coli*
Morin (333.33–1000.00 µg/mL)	-	-	-	-
Control (morin)	-	-	-	-
CH (2 mg/mL)	-	-	-	-
CH (3.33 mg/mL)	7.0 ± 1.87	-	-	-
CH (5 mg/mL)	15.0 ± 2.16	-	12.0 ± 0.80	-
CH (10 mg/mL)	16.3 ± 1.09	-	15.0 ± 1.28	-
Control (CH)	10.0 ± 0.35	-	12.0 ± 1.56	-
LGN (10 mg/mL)	12.0 ± 0.92	-	-	-
LGN (50 mg/mL)	13.5 ± 1.5	-	-	-
LGN (66.6 mg/mL)	14.3 ± 1.67	-	-	-
LGN (100 mg/mL)	17.8 ± 1.34	-	-	-
Control (LGN)	-	-	-	-
CH–morin	15.5 ± 2.5	15.0 ± 2.74	-	-
Control (CH–morin)	-	-	-	-
LGN–morin	18.5 ± 0.5	16 ± 0.82	-	-
Control (LGN–morin)	-	-	-	-
CH–LGN	18.5 ± 1.5	13.0 ± 1.26		
CH–LGN–morin	15.7 ± 1.69	14.0 ± 1.05	15.0 ± 1.23	14.0 ± 0.88
Control (CH–LGN–H_2_O)	15.0 ± 1.3	10.0 ± 1.00	-	-
Control (gentamicin)	23.1 ± 1.01	21 ± 1.32	20.0 ± 0.65	23 ± 0.42

**Table 2:** Compositions of the studied single-, two- and three-component systems.

System	Composition/Concentration
Single-component systems	
Morin (in EtOH:PBS (pH = 4.6) = 1:1) Negative control	333.30; 500.00; 700.00; 1000.00 µg/mL EtOH:PBS (pH = 4.6) = 1:1, *v*/*v*
CH (in 1% LA) Negative control	2.00; 3.33; 5.00; 10.00 mg/mL 1% LA
LGN (in distilled water) Negative control	10.00; 50.00; 66.66; 100.00 mg/mL Distilled water
Two-component systems	
CH–morin Negative control	CH (10.00 mg/mL):morin (1000.00 µg/mL) = 1:1, *v*/*v* LA:EtOH:PBS (pH = 4.6) = 1:0.5:0.5 *v*/*v*/*v*
LGN–morin Negative control	LGN (200.00 mg/mL):morin (1000.00 µg/mL) = 1:1, *v*/*v* H_2_O:EtOH:PBS (pH = 4.6) = 1:0.5:0.5, *v*/*v*/*v*
CH–LGN	CH (10.00 mg/mL):LGN (200.00 mg/mL) = 1:1, *v*/*v*
Three-component system	
Morin–CH–LGN Negative control	Morin (1000.00 µg/mL):CH (10.00 mg/mL):LGN (200.00 mg/mL) = 1:1:1, *v*/*v*/*v* CH (10.00 mg/mL):LGN (200.00 mg/mL):H_2_O = 1:1:1, *v*/*v*/*v*

CH—chitosan; LGN—lignin; LA—lactic acid.

## Data Availability

Not applicable.
